# Impaired glymphatic system in genetic frontotemporal dementia: a GENFI study

**DOI:** 10.1093/braincomms/fcae185

**Published:** 2024-06-14

**Authors:** Enrico Premi, Matteo Diano, Irene Mattioli, Daniele Altomare, Valentina Cantoni, Martina Bocchetta, Roberto Gasparotti, Emanuele Buratti, Marta Pengo, Arabella Bouzigues, Lucy L Russell, Phoebe H Foster, Eve Ferry-Bolder, Carolin Heller, John C van Swieten, Lize C Jiskoot, Harro Seelaar, Fermin Moreno, Raquel Sanchez-Valle, Daniela Galimberti, Robert Laforce, Caroline Graff, Mario Masellis, Maria Carmela Tartaglia, James B Rowe, Elizabeth Finger, Rik Vandenberghe, Alexandre de Mendonça, Chris R Butler, Alexander Gerhard, Simon Ducharme, Isabelle Le Ber, Pietro Tiraboschi, Isabel Santana, Florence Pasquier, Matthis Synofzik, Johannes Levin, Markus Otto, Sandro Sorbi, Jonathan D Rohrer, Barbara Borroni, Coinvestigators GENFI Consortium, David L Thomas, Emily Todd, Caroline V Greaves, Jennifer Nicholas, Kiran Samra, Rhian Convery, Carolyn Timberlake, Thomas Cope, Timothy Rittman, Andrea Arighi, Chiara Fenoglio, Elio Scarpini, Giorgio Fumagalli, Vittoria Borracci, Giacomina Rossi, Giorgio Giaccone, Giuseppe Di Fede, Paola Caroppo, Sara Prioni, Veronica Redaelli, David Tang-Wai, Ekaterina Rogaeva, Miguel Castelo-Branco, Morris Freedman, Ron Keren, Sandra Black, Sara Mitchell, Christen Shoesmith, Robart Bartha, Rosa Rademakers, Jackie Poos, Janne M Papma, Lucia Giannini, Rick van Minkelen, Yolande Pijnenburg, Benedetta Nacmias, Camilla Ferrari, Cristina Polito, Gemma Lombardi, Valentina Bessi, Michele Veldsman, Christin Andersson, Hakan Thonberg, Linn Öijerstedt, Vesna Jelic, Paul Thompson, Tobias Langheinrich, Albert Lladó, Anna Antonell, Jaume Olives, Mircea Balasa, Nuria Bargalló, Sergi Borrego-Ecija, Ana Verdelho, Carolina Maruta, Tiago Costa-Coelho, Gabriel Miltenberger, Alazne Gabilondo, Ana Gorostidi, Jorge Villanua, Marta Cañada, Mikel Tainta, Miren Zulaica, Myriam Barandiaran, Patricia Alves, Benjamin Bender, Carlo Wilke, Lisa Graf, Annick Vogels, Mathieu Vandenbulcke, Philip Van Damme, Rose Bruffaerts, Koen Poesen, Pedro Rosa-Neto, Serge Gauthier, Agnès Camuzat, Alexis Brice, Anne Bertrand, Aurélie Funkiewiez, Daisy Rinaldi, Dario Saracino, Olivier Colliot, Sabrina Sayah, Catharina Prix, Elisabeth Wlasich, Olivia Wagemann, Sandra Loosli, Sonja Schönecker, Tobias Hoegen, Jolina Lombardi, Sarah Anderl-Straub, Adeline Rollin, Gregory Kuchcinski, Maxime Bertoux, Thibaud Lebouvier, Vincent Deramecourt, Beatriz Santiago, Diana Duro, Maria João Leitão, Maria Rosario Almeida, Miguel Tábuas-Pereira, Sónia Afonso

**Affiliations:** Stroke Unit, ASST Spedali Civili Brescia, Brescia, 25123, Italy; Department of Psychology, University of Torino, Turin, 10124, Italy; Department of Clinical and Experimental Sciences, University of Brescia, Brescia, 25123, Italy; Department of Clinical and Experimental Sciences, University of Brescia, Brescia, 25123, Italy; Department of Clinical and Experimental Sciences, University of Brescia, Brescia, 25123, Italy; Dementia Research Centre, Department of Neurodegenerative Disease, UCL Queen Square Institute of Neurology, London, WC1N, UK; Centre for Cognitive and Clinical Neuroscience, Division of Psychology, Department of Life Sciences, College of Health, Medicine and Life Sciences, Brunel University London, London, UB8 3PN, UK; Neuroradiology Unit, University of Brescia, Brescia, 25123, Italy; International Centre for Genetic Enginneering and Biotechnology, Trieste, 34149, Italy; Department of Clinical and Experimental Sciences, University of Brescia, Brescia, 25123, Italy; Dementia Research Centre, Department of Neurodegenerative Disease, UCL Queen Square Institute of Neurology, London, WC1N, UK; Dementia Research Centre, Department of Neurodegenerative Disease, UCL Queen Square Institute of Neurology, London, WC1N, UK; Dementia Research Centre, Department of Neurodegenerative Disease, UCL Queen Square Institute of Neurology, London, WC1N, UK; Dementia Research Centre, Department of Neurodegenerative Disease, UCL Queen Square Institute of Neurology, London, WC1N, UK; Dementia Research Centre, Department of Neurodegenerative Disease, UCL Queen Square Institute of Neurology, London, WC1N, UK; Department of Neurology, Erasmus Medical Centre, Rotterdam, 2040 3000, The Netherlands; Department of Neurology, Erasmus Medical Centre, Rotterdam, 2040 3000, The Netherlands; Department of Neurology, Erasmus Medical Centre, Rotterdam, 2040 3000, The Netherlands; Cognitive Disorders Unit, Department of Neurology, Donostia University Hospital, San Sebastian, 20014, Spain; Neuroscience Area, Biodonostia Health Research Institute, San Sebastian, Gipuzkoa, 20014, Spain; Alzheimer’s Disease and Other Cognitive Disorders Unit, Neurology Service, Hospital Clínic, Institut d’Investigacións Biomèdiques August Pi I Sunyer, University of Barcelona, Barcelona, 08036, Spain; Fondazione Ca’ Granda, IRCCS Ospedale Policlinico, Milan, 20122, Italy; Centro Dino Ferrari, University of Milan, Milan, 20122, Italy; Clinique Interdisciplinaire de Mémoire, Département des Sciences Neurologiques, CHU de Québec, Faculté de Médecine, Université Laval, Quebec City, G1V 0A6, Canada; Center for Alzheimer Research, Division of Neurogeriatrics, Department of Neurobiology, Care Sciences and Society, Bioclinicum, Karolinska Institutet, Solna, 17177, Sweden; Unit for Hereditary Dementias, Theme Aging, Karolinska University Hospital, Solna, 17177, Sweden; Sunnybrook Health Sciences Centre, Sunnybrook Research Institute, University of Toronto, Toronto, ON M4N 3M5, Canada; Tanz Centre for Research in Neurodegenerative Diseases, University of Toronto, Toronto, ON M4N 3M5, Canada; Department of Clinical Neurosciences, University of Cambridge, Cambridge, CB2 1TN, UK; Department of Clinical Neurological Sciences, University of Western Ontario, London, ON N6A 5A5, Canada; Laboratory for Cognitive Neurology, Department of Neurosciences, KU Leuven, Leuven, 3000, Belgium; Neurology Service, University Hospitals Leuven, Leuven, 3000, Belgium; Leuven Brain Institute, KU Leuven, Leuven, 3000, Belgium; Faculty of Medicine, University of Lisbon, Lisbon, 1649-004, Portugal; Nuffield Department of Clinical Neurosciences, Medical Sciences Division, University of Oxford, Oxford, OX1 4BH, UK; Department of Brain Sciences, Imperial College London, London, SW7 2BX, UK; Division of Neuroscience and Experimental Psychology, Wolfson Molecular Imaging Centre, University of Manchester, Manchester, M13 9GB, UK; Department of Geriatric Medicine, University of Duisburg-Essen, Duisburg, 47057, Germany; Department of Nuclear Medicine, University of Duisburg-Essen, Duisburg, 47057, Germany; Department of Psychiatry, McGill University Health Centre, McGill University, Montreal, H3H 2R9, Québec, Canada; McConnell Brain Imaging Centre, Montreal Neurological Institute, McGill University, Montreal, H3H 2R9, Québec, Canada; Sorbonne Université, Paris Brain Institute—Institut du Cerveau—ICM, Inserm U1127, CNRS UMR 7225, Paris, 75013, France; Centre de Référence des Démences Rares ou Précoces, IM2A, Département de Neurologie, AP-HP - Hôpital Pitié-Salpêtrière, Paris, 75651, France; Département de Neurologie, AP-HP - Hôpital Pitié-Salpêtrière, Paris, 5783, France; Fondazione IRCCS Istituto Neurologico Carlo Besta, Milan, 20133, Italy; Neurology Service, Faculty of Medicine, University Hospital of Coimbra (HUC), University of Coimbra, Coimbra, 3000-214, Portugal; Center for Neuroscience and Cell Biology, Faculty of Medicine, University of Coimbra, Coimbra, 3000-214, Portugal; University of Lille, Lille, 59000, France; Inserm 1172, Lille, Lille, 59000, France; CHU, CNR-MAJ, Labex Distalz, LiCEND Lille, Lille, 59000, France; Division Translational Genomics of Neurodegenerative Diseases, Hertie-Institute for Clinical Brain Research and Center of Neurology, University of Tübingen, Tübingen, 72074, Germany; Center for Neurodegenerative Diseases (DZNE), Tübingen, 72076, Germany; Department of Neurology, Ludwig-Maximilians Universität München, Munich, 80539, Germany; German Center for Neurodegenerative Diseases (DZNE), Munich, 81377, Germany; Munich Cluster of Systems Neurology (SyNergy), Munich, 81377, Germany; Department of Neurology, University of Ulm, Ulm, 89081, Germany; Department of Neurofarba, University of Florence, Florence, 50139, Italy; IRCCS Fondazione Don Carlo Gnocchi, Florence, 50124, Italy; Dementia Research Centre, Department of Neurodegenerative Disease, UCL Queen Square Institute of Neurology, London, WC1N, UK; Department of Clinical and Experimental Sciences, University of Brescia, Brescia, 25123, Italy; Department of Continuity of Care and Frailty, ASST Spedali Civili Brescia, Brescia, 25123, Italy

**Keywords:** frontotemporal dementia, frontotemporal lobar degeneration, glymphatic system, DTI-ALPS, genetic

## Abstract

The glymphatic system is an emerging target in neurodegenerative disorders. Here, we investigated the activity of the glymphatic system in genetic frontotemporal dementia with a diffusion-based technique called diffusion tensor image analysis along the perivascular space. We investigated 291 subjects with symptomatic or presymptomatic frontotemporal dementia (112 with *chromosome 9 open reading frame 72* [*C9orf72*] expansion, 119 with *granulin* [*GRN*] mutations and 60 with *microtubule-associated protein tau* [*MAPT*] mutations) and 83 non-carriers (including 50 young and 33 old non-carriers). We computed the diffusion tensor image analysis along the perivascular space index by calculating diffusivities in the *x*-, *y*- and *z*-axes of the plane of the lateral ventricle body. Clinical stage and blood-based markers were considered. A subset of 180 participants underwent cognitive follow-ups for a total of 640 evaluations. The diffusion tensor image analysis along the perivascular space index was lower in symptomatic frontotemporal dementia (estimated marginal mean ± standard error, 1.21 ± 0.02) than in old non-carriers (1.29 ± 0.03, *P* = 0.009) and presymptomatic mutation carriers (1.30 ± 0.01, *P* < 0.001). In mutation carriers, lower diffusion tensor image analysis along the perivascular space was associated with worse disease severity (*β* = −1.16, *P* < 0.001), and a trend towards a significant association between lower diffusion tensor image analysis along the perivascular space and higher plasma neurofilament light chain was reported (*β* = −0.28, *P* = 0.063). Analysis of longitudinal data demonstrated that worsening of disease severity was faster in patients with low diffusion tensor image analysis along the perivascular space at baseline than in those with average (*P* = 0.009) or high (*P* = 0.006) diffusion tensor image analysis along the perivascular space index. Using a non-invasive imaging approach as a proxy for glymphatic system function, we demonstrated glymphatic system abnormalities in the symptomatic stages of genetic frontotemporal dementia. Such measures of the glymphatic system may elucidate pathophysiological processes in human frontotemporal dementia and facilitate early phase trials of genetic frontotemporal dementia.

## Teaser

Premi *et al*. investigated the activity of the glymphatic system in genetic frontotemporal dementia using a proxy measure with a diffusion-based MRI technique. The authors demonstrated that glymphatic system is impaired in symptomatic stages of frontotemporal dementia and baseline scores of glymphatic system assessment predicted clinical progression.

## Graphical Abstract

**Graphical Abstract:**
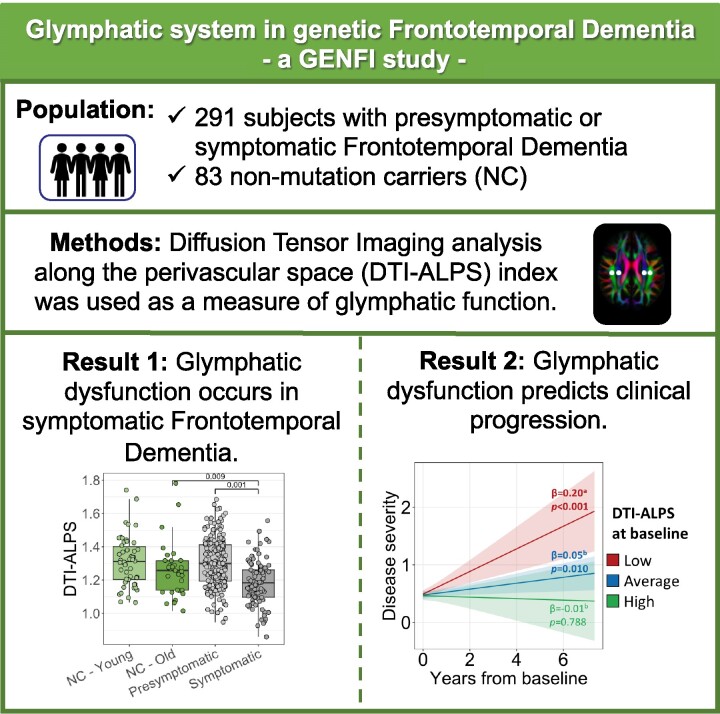

## Introduction

Frontotemporal dementia is marked by changes in behaviour, language and executive control, in association with multiple neuropathological substrates and heterogeneous genetic background.^1-3^

Clinical phenotypes mainly encompass behavioural-variant (bv) frontotemporal dementia and primary progressive aphasia (PPA).^1,2^ The pathogenic mechanisms of frontotemporal dementia are determined by intracellular accumulation of aberrant proteins, including mainly tau [frontotemporal lobar degeneration-tau (FTLD-tau)] or TAR DNA–binding protein 43 (TDP-43, FTLD-TDP).^4,5^ Around 30% of frontotemporal dementia is familial, most commonly caused by autosomal dominant genetic mutations within *microtubule-associated protein tau* (*MAPT*), *granulin* (*GRN*) and *chromosome 9 open reading frame 72* (*C9orf72*) genes.^6^

Genetic frontotemporal dementia cases represent a privileged scenario to study the earliest phases of the disease in presymptomatic individuals and assess the changes in defined neuropathological subtypes, such as FTLD-tau, associated with *MAPT* mutations, or FTLD-TDP, due to *GRN* or *C9orf72* genetic variations.^7^

Impairment of the glymphatic function, the brain waste clearing system,^8^ has recently been suggested to play a role in several neurodegenerative disorders.^9-14^ The glymphatic pathway promotes the flow of CSF into the brain along arterial perivascular spaces and subsequently into the brain interstitium through an astrocytic aquaporin-4-dependent mechanism. The pathway then directs the CSF flow towards the venous perivascular and perineuronal spaces, ultimately clearing solutes into meningeal and cervical lymphatic drainage vessels.^15,16^

Despite the expanding concepts on glymphatic physiology and pathology, the *in vivo* assessment of the glymphatic function is still hampered by the need of non-invasive imaging techniques for its quantification.^17^ Recent studies have demonstrated the possibility of measuring glymphatic functions using MRI.^18^ In particular, the diffusion tensor image analysis along the perivascular space (DTI-ALPS) technique has been utilized to estimate the efficiency of the glymphatic system by the DTI-ALPS-index, as a measure of perivascular clearance activity in the human brain.^18^ This index showed its significant consistency with the classical detection clearance rate calculated on glymphatic MRI after intrathecal administration of gadolinium.^19^

DTI-ALPS is impaired in several neurological conditions.^10-13,17,20,21^ More recently, significantly decreased DTI-ALPS values were reported in bv frontotemporal dementia,^14^ and in agreement with these findings, glymphatic dysfunction was associated with accumulation of tau and TDP-43 proteins in animal models.^22-24^

However, a few questions still need to be addressed. It is not yet known whether the glymphatic dysfunction (i) is an early event in frontotemporal dementia, already detectable in presymptomatic disease stages; (ii) is associated with a specific FTLD proteinopathy or may represent a common pathway related to neurodegeneration; (iii) is a marker of disease severity; (iv) correlates with markers of neuronal dysfunction or axonal damage, such as plasma neurofilament light chain (NfL) or glial fibrillary acidic protein (GFAP)^25,26^; and/or (v) predicts disease progression over time.

These questions prompted the current cross-sectional and longitudinal study. Our specific aim was to evaluate glymphatic system abnormalities in presymptomatic and symptomatic individuals with pathogenic mutations within the *MAPT*, *GRN* and *C9orf72* genes, to provide novel insight into the pathophysiology of genetic frontotemporal dementia.

## Materials and methods

### Participants

From the GENFI cohort study, individuals carrying a pathogenic frontotemporal dementia variation and their non-carrier (NC) family members were recruited from research centres across Europe and Canada (www.genfi.org.uk). Inclusion and exclusion criteria have been previously described.^27^ Here, we considered a consecutive sample of 374 participants recruited from 30 January 2012 to 30 January 2021. Among them, 112 were *C9orf72* expansion carriers (68 presymptomatic and 44 symptomatic), 119 were *GRN* mutation carriers (88 presymptomatic and 31 symptomatic), 60 were *MAPT* mutation carriers (43 presymptomatic and 17 symptomatic), and 83 were mutation NC individuals recruited among siblings.

All participants underwent the GENFI standardized assessment at enrollment.^7^ During the first visit, demographic information was collected. The years to expected onset were calculated as the difference between age at assessment and mean age at onset within the family, as previously described.^28^ We assessed disease stage using the Clinical Dementia Rating (CDR)® Dementia Staging Instrument plus National Alzheimer’s Coordinating Centre (NACC) behaviour and language domains (CDR® plus NACC FTLD),^29^ hereinafter referred to as CDR-FTLD.

Mutation carriers were divided into two disease stages based on their global CDR-FTLD score: presymptomatic (0 or 0.5) and fully symptomatic (1 or more).

Among the 291 mutation carriers, 282 participants underwent CDR-FTLD at baseline. A subgroup of mutation carriers also underwent CDR-FTLD at different follow-ups (180 with at least two evaluations, 105 with at least three, 55 with at least four, 15 with at least five and 3 with at least six), for a total of 640 evaluations.

Local ethics committees approved the study at each site, and all participants provided written informed consent; the study was conducted according to the Declaration of Helsinki.

### MRI acquisition

MRI protocol was common to all the GENFI sites and adapted for different scanners (see Supplementary Table 1 for the scanner list); no pre-study phantom harmonization was performed at local level. Each subject underwent a 3 T MRI at their local site, which have scanners from three different manufacturers (Philips Healthcare, GE Healthcare Life Sciences and Siemens Healthcare Diagnostic). The protocol included a volumetric T_1_-weighted (magnetization-prepared rapid gradient echo) scan and a diffusion-weighted imaging scan (consisting of with either four or five b0 images and 61 diffusion-weighted images or 64 diffusion-weighted images both with a *b*-value of 1000 s/mm^2^, depending on the different acquisition sites), as previously reported.^7,27^

### MRI preprocessing and analyses

#### Structural grey matter data

T_1_-weighted images were processed with the voxel-based morphometry pipeline implemented in the Computational Anatomy Toolbox (CAT12 v.1742) (www.neuro.uni-jena.de/cat) for SPM12 (SPM12 v.7219) (www.fil.ion.ucl.ac.uk/spm/software/spm12) running on MATLAB R2019b (the MathWorks, Inc., Natick, MA, USA). The voxel-based morphometry pipeline steps consist of tissue segmentation, spatial normalization to a standard Montreal National Institute template, modulation and smoothing.^30^ The pipeline was also used to calculate the total intracranial volumes for each subject.

#### Diffusion-weighted data

Diffusion-weighted imaging scans were preprocessed using Mrtrix3 and FSL software.^31^ Diffusion-weighted imaging scans were analysed using the FDT tool of the FMRIB Software Library (FSL, v6.0). Diffusion-weighted imaging data were denoised and corrected for eddy current–induced distortions and subject movements. These slices are replaced by non-parametric predictions by Gaussian process. Diffusion tensor measures of fractional anisotropy (FA) and mean diffusivity were calculated running DTIFIT script, which fits a diffusion tensor model at each voxel, and subsequently registered to the study template.

#### DTI-ALPS index computation

The DTI-ALPS method is based on the assumption that the perivascular interstitial fluid movement in the white matter at the level of the lateral ventricle body is dominant along the parallelly aligned medullary veins (*x*-axis), which run perpendicular to the ventricular wall; the *y*-axis is given by projection fibres running in the head–foot direction, mainly adjacent to the lateral ventricle, and the *z*-axis represents association fibres, running in the anterior–posterior direction outside the projection fibres.^18^ In the projection area, dominant fibres run in the *z*-axis direction, perpendicular to both the *x*- and *y*-axes, whereas in the association area, dominant fibres run in the *y*-axis direction, perpendicular to both the *x*- and *z*-axes. The DTI-ALPS index is calculated from the diffusivity in each direction of the projection and association fibre regions at the lateral ventricular body level and estimates the effect of the glymphatic system impairment on the diffusivity along the perivascular space of medullary veins. In calculating the DTI-ALPS index, the FA maps of each subject were registered linearly first and nonlinearly subsequently into the high-resolution FSL_HCP1065_FA standard space image. Spherical regions of interest (ROIs) measuring 5 mm in radius were placed *a priori* in the projection and association areas at the level of the lateral ventricle bodies in the left and right hemispheres onto the same FA template (see Supplementary Fig. 1). The ROIs were then automatically registered to the subjects’ FA map, and all subjects underwent visual inspection (by mean diffusivity and by an expert neurologist [initials: IM]) to check correct ROI placement. If ROIs were not correctly placed, due to artefacts or poor image quality, the subject was excluded from the study (*n* = 57, 14.3%). Finally, in line with the previous literature data, three DTI-ALPS indices were calculated using the following formula^18,32^:


$$\mathrm{DTI}\;\text{-}\;\mathrm{ALPS}=\frac{\mathrm{mean}(\mathrm{Dxx}\;\mathrm{proj},\;\mathrm{Dxx}\;\mathrm{assoc})}{\mathrm{mean}(\mathrm{Dyy}\;\mathrm{proj},\;\mathrm{Dzz}\;\mathrm{assoc})}.$$

The left and right DTI-ALPS (L-DTI-ALPS and R-DTI-ALPS) indices were calculated as a ratio of the mean of the *x*-axis diffusivity in the projection area (Dxx, proj) and *x*-axis diffusivity in the association area (Dxx, assoc) to the mean of the *y*-axis diffusivity in the projection area (Dyy, proj) and the *z*-axis diffusivity in the association area (Dzz, assoc) on each hemisphere, respectively.

The bilateral mean DTI-ALPS index (hereinafter referred to as DTI-ALPS) was calculated as the average of the R-DTI-ALPS and L-DTI-ALPS.

A DTI-ALPS index close to 1.0 reflects lower diffusivity with greater glymphatic system impairment, whereas higher values indicate greater diffusivity and less glymphatic system impairment. In all the analyses, the bilateral mean DTI-ALPS index has been used as primary measure.^18^

### Plasma NfL and plasma GFAP quantification

In a subset of participants, at the time of clinical assessment, plasma was collected by venepuncture and centrifuged (2000 *g*, 10 min, at room temperature), according to GENFI protocol.^33^ Serum was frozen at −80°C within 3 h after collection, shipped and analysed without any previous thaw–freeze cycle. Samples were measured using the multiplex Neurology 4-Plex A kit (102153, Quanterix, Lexington, KY, USA) on the SIMOA HD-1 Analyser following the manufacturer’s instructions, as previously published.^33^ The lower limit of detection of the assay for NfL and GFAP was 0.104 and 0.221 pg/mL, respectively. Quality control samples had a mean intra-assay and inter-assay coefficient of variation of <10%. Technicians were blinded to the genotypic and clinical status of the samples. Plasma NfL and GFAP measures were available for 284 and 235 participants, respectively.

### Statistical analyses

Since symptomatic individuals are older than the presymptomatic ones and to avoid biases due to the comparisons of groups with different age, we stratified the NC group into two subgroups based on the mean age (49 ± 13 years): young NC (40 ± 6 years) and old NC (62 ± 7 years). Moreover, due to the large number of pairwise comparisons, we focused only on pre-defined comparisons of interest: young NC versus presymptomatic mutation carriers, old NC versus symptomatic mutation carriers and presymptomatic versus symptomatic within the same genetic mutation groups. Differences among groups in the sociodemographic and clinical features were assessed using Kruskal–Wallis rank sum tests for continuous variables or tests for equality of proportions for categorical variables.

Associations between DTI-ALPS and other variables were assessed using linear models adjusted for covariates, namely age, sex and MRI site. When plasma markers were considered, site where samples were analysed was added as covariate as well.

In order to assess the association between DTI-ALPS at baseline and longitudinal change in disease severity (CDR-FTLD), baseline DTI-ALPS scores of mutation carriers were converted into *Z*-scores using mean and standard deviation of NC (both the young and the old ones), and mutation carriers were classified as having ‘high’ (*Z*-score > 1), ‘average’ (*Z*-score between −1 and 1) or ‘low’ (*Z*-score < −1) baseline DTI-ALPS. Subsequently, the association between DTI-ALPS at baseline and longitudinal change in disease severity was assessed using a linear mixed model with longitudinal CDR-FTLD as the dependent variable; DTI-ALPS groups (i.e. ‘high’, ‘average’ and ‘low’), time (years) from baseline and their interaction as independent variables; and age, sex, CDR-FTLD at baseline and site as covariates; random intercepts and slopes at the subject level were computed.

When the number of groups in the comparison was larger than two, *post hoc* pairwise comparisons were adjusted using the false discovery rate correction. Significance was set at *P* < 0.05. All statistical analyses were performed with R, version 4.3.0 (The R Project for Statistical Computing, https://www.r-project.org/).

## Results

### Participants

Table 1 illustrates demographic and clinical features of the 374 study participants. As expected, presymptomatic mutation carriers (mean age: 39–45 years; mean CDR-FTLD score: 0.1–0.2) were younger and had better global cognition and functioning than symptomatic mutation carriers (mean age: 59–62 years, *P* < 0.05; mean CDR-FTLD score: 1.7–2.0, *P* < 0.05). In presymptomatic mutation carriers, the expected age at onset was similar in the three genetic groups (*P* = 0.446). In symptomatic mutation carriers, bv frontotemporal dementia was the most prevalent clinical phenotype.

**Table 1:** Demographic and clinical features of the study participants and DTI-ALPS values disaggregating by genetic mutation and disease stage

Features	NC young (*n* = 50)	NC old (*n* = 33)	Presymptomatic			Symptomatic			*P*-value
			*C9orf72* (*n* = 68)	*GRN* (*n* = 88)	*MAPT* (*n* = 43)	*C9orf72* (*n* = 44)	*GRN* (*n* = 31)	*MAPT* (*n* = 17)	
Age (years)	40 (6)	62 (7)	44 (11)	45 (12) a	39 (9)	62 (8) c	62 (9) c	59 (10) c	**<0**.**001**
Sex, female % (*n*)	58 (29)	61 (20)	59 (40)	68 (60)	56 (24)	34 (15)	42 (13)	35 (6)	**0**.**006**
Expected age at onset, years	-	-	−16 (11)	−15 (12)	−13 (10)	-	-	-	0.446
Education (years)	15 (3)	14 (3)	14 (3)	15 (4)	15 (3)	13 (3) c	12 (3) c	15 (3)	**<0**.**001**
Phenotype, bv frontotemporal dementia %	-	-	-	-	-	64 (28)	55 (17)	76 (13)	0.330
Phenotype, PPA %	-	-	-	-	-	5 (2)	35 (11)	6 (1)	**<0**.**001**
Phenotype, unclassified frontotemporal dementia %	-	-	-	-	-	32 (14)	10 (3)	18 (3)	0.066
CDR-FTLD	0.1 (0.2)	0.1 (0.2)	0.1 (0.2)	0.1 (0.2)	0.2 (0.3)	1.8 (0.9) b, c	2.0 (0.9) b, c	1.7 (0.9) b, c	**<0**.**001**
TIV	1449 (139)	1458 (162)	1450 (146)	1443 (144)	1473 (148)	1481 (151)	1426 (152)	1406 (172)	0.466
Biological markers									
NfL (pg/mL)	8.84 (11.46)	13.72 (6.87)	12.76 (16.20) a	8.70 (4.78)	7.21 (2.98)	43.87 (31.58) b, c	77.05 (40.12) b, c	22.82 (10.79) c	**<0**.**001**
GFAP (pg/mL)	73.57 (26.20)	140.94 (36.32)	104.85 (60.78)	94.82 (48.25)	74.82 (28.03)	162.16 (58.94) c	290.29 (358.69) c	148.04 (95.43) c	**<0**.**001**
DTI-ALPS index									
DTI-ALPS	1.32 (0.02)	1.29 (0.03)	1.28 (0.02)	1.32 (0.02)	1.28 (0.03)	1.19 (0.03) b, c	1.24 (0.03) c	1.21 (0.04)	**0**.**001**
Left DTI-ALPS	1.32 (0.02)	1.29 (0.03)	1.28 (0.02)	1.33 (0.02)	1.28 (0.03)	1.19 (0.03) b, c	1.22 (0.03) c	1.24 (0.04)	**0**.**003**
Right DTI-ALPS	1.32 (0.02)	1.30 (0.03)	1.28 (0.02)	1.32 (0.02)	1.27 (0.03)	1.20 (0.03)	1.26 (0.03)	1.19 (0.04)	**0**.**009**

Values are means (standard deviation) for continuous variables or percentages (raw numbers) for categorical variables, unless otherwise specified. DTI-ALPS values are estimated marginal means (standard errors).

DTI-ALPS, diffusion tensor image analysis along the perivascular space; NC, non-carriers; *C9orf72*, *chromosome 9 open reading frame 72*; *GRN*, *granulin*; *MAPT*, *microtubule-associated protein tau*; bv, behavioural-variant; CDR-FTLD, CDR® Dementia Staging Instrument plus NACC behaviour and language domains; TIV, total intracranial volume; NfL, neurofilament light chain; GFAP, glial fibrillary acidic protein.

^a^Significant *post hoc* comparisons (false discovery rate correction) of presymptomatic mutation carriers versus young NC are reported.

^b^Significant *post hoc* comparisons (false discovery rate correction) of symptomatic mutation carriers versus old NC are reported.

^c^Significant *post hoc* comparisons (false discovery rate correction) of presymptomatic versus symptomatic mutation carriers within the same mutation are reported.

### Glymphatic system according to clinical, genetic and neuropathological features

**Figure 1:**
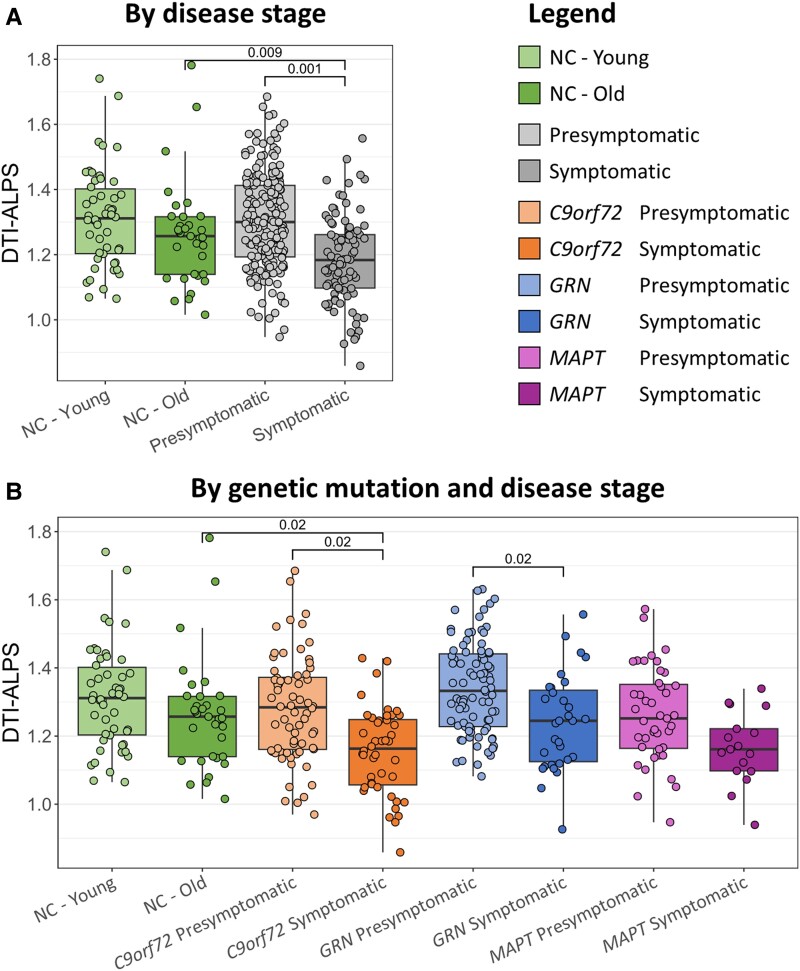
**Distribution of DTI-ALPS across disaggregating by disease stage (A) or by genetic mutation and disease stage (B).** DTI-ALPS, diffusion tensor image analysis along the perivascular space. Comparisons of interest were ‘young NC versus presymptomatic’, ‘old NC versus symptomatic’ and ‘presymptomatic versus symptomatic’ in **A** (i.e. three pairwise comparisons in total) and ‘young NC versus all presymptomatic groups’, ‘old NC versus all symptomatic groups’ and ‘presymptomatic versus symptomatic within the same mutation groups’ within the three genetic groups in **B** (i.e. nine pairwise comparisons in total). Statistics values: *F*(3) = 6.05, *P* < 0.001 for **A** and *F*(7) = 3.46, *P* = 0.001 for **B**. *Post hoc* pairwise comparisons of interest were adjusted using the false discovery rate correction.

Figure 1 shows the distribution of DTI-ALPS according to disease stage and genetic group.

The DTI-ALPS index was lower in symptomatic mutation carriers (estimated marginal mean ± standard error, 1.21 ± 0.02) than in old NC (1.29 ± 0.03, *P* = 0.009) and presymptomatic mutation carriers (1.30 ± 0.01, *P* = 0.001), denoting glymphatic impairment in symptomatic frontotemporal dementia (Fig. 1A).

Disaggregating also by genetic group, DTI-ALPS was lower in symptomatic *C9orf72* expansion carriers (1.19 ± 0.03) than in old NC (1.29 ± 0.03, *P* = 0.020) and in presymptomatic *C9orf72* mutation carriers (1.28 ± 0.02, *P* = 0.020) and in symptomatic *GRN* mutation carriers (1.24 ± 0.03) than in presymptomatic *GRN* mutation carriers (1.32 ± 0.02, *P* = 0.020), with a similar yet not statistically significant trend also in *MAPT* mutation carriers (1.21 ± 0.04 in symptomatic versus 1.28 ± 0.03 in presymptomatic) (Fig. 1B and Table 1).

As reported in Table 1, similar results were found when L-DTI-ALPS and R-DTI-ALPS were considered instead of the global DTI-ALPS.

No significant differences in the DTI-ALPS index were found when comparing patients with bv frontotemporal dementia (1.20 ± 0.02), PPA (1.23 ± 0.04) and those with unclassified frontotemporal dementia (1.18 ± 0.04, *P* = 0.604), nor in the L-DTI-ALPS index (bv frontotemporal dementia: 1.21 ± 0.03; PPA: 1.23 ± 0.05; unclassified frontotemporal dementia: 1.19 ± 0.05, *P* = 0.769) or R-DTI-ALPS index (bv frontotemporal dementia: 1.20 ± 0.03; PPA: 1.24 ± 0.05; unclassified frontotemporal dementia: 1.17 ± 0.04, *P* = 0.567).

No significant differences in the DTI-ALPS index were found when comparing FTLD-TDP (symptomatic *C9orf72* and *GRN* mutation carriers, 1.20 ± 0.02) to FTLD-tau (symptomatic *MAPT* mutation carriers, 1.19 ± 0.04, *P* = 0.819).

### Associations between glymphatic system and disease severity, expected years of onset and plasma biomarkers

**Figure 2:**
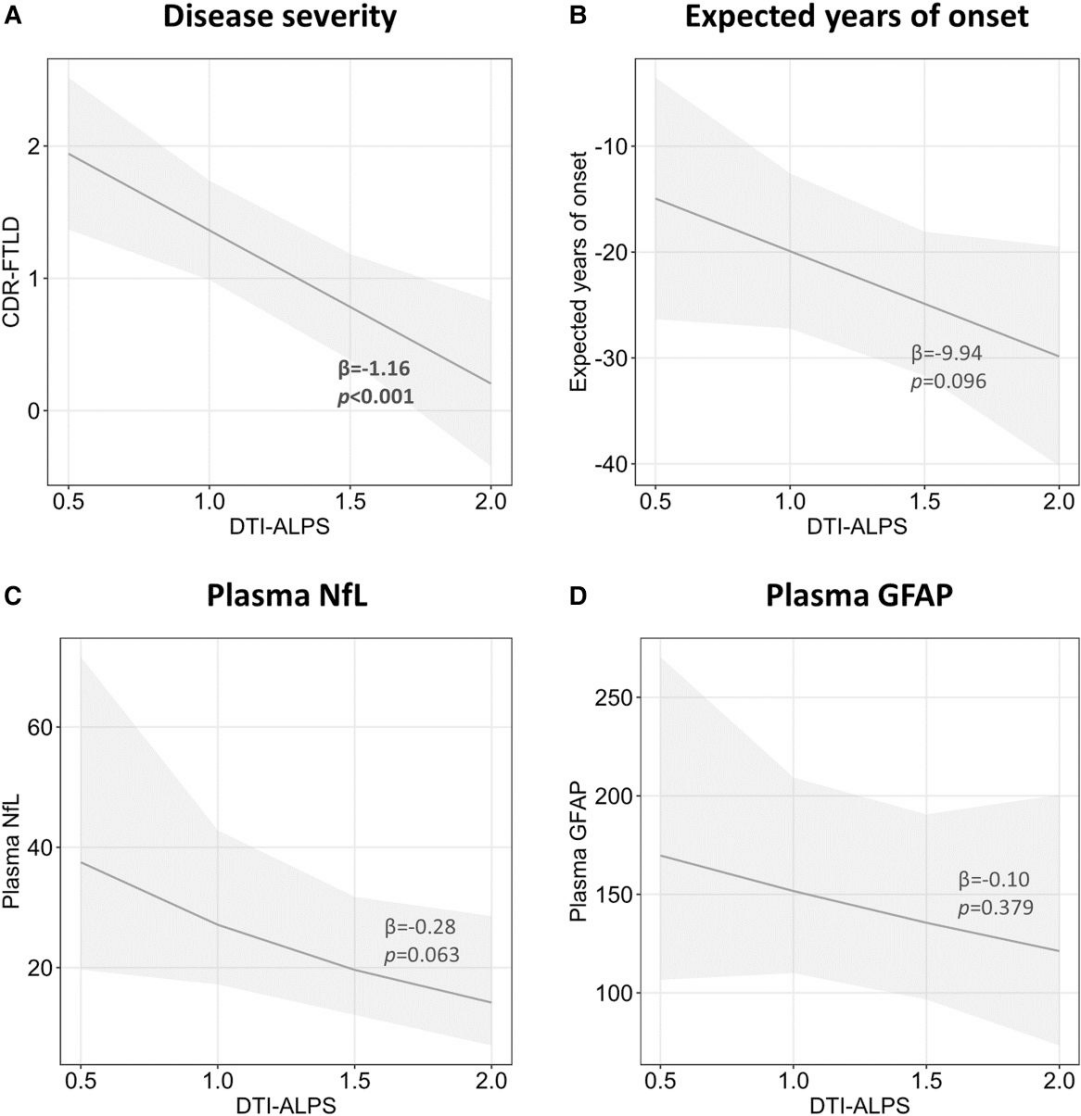
**Association of DTI-ALPS with disease severity (i.e. CDR-FTLD) (A), expected years of onset (B), plasma NfL (C) and plasma GFAP (D) in mutation carriers.** DTI-ALPS, diffusion tensor image analysis along the perivascular space; CDR-FTLD, CDR® Dementia Staging Instrument plus NACC behaviour and language domains; NfL, neurofilament light; GFAP, glial fibrillary acidic protein. Plasma NfL and GFAP values were log-transformed. Statistics values: *F*(1) = 12.48, *P* < 0.001 for **A**; *F*(1) = 2.80, *P* = 0.096 for **B**; *F*(1) = 3.49, *P* = 0.063 for **C**; and *F*(1) = 0.78, *P* = 0.379 for **D**.

In all mutation carriers combined, lower DTI-ALPS was associated with worse disease severity (CDR-FTLD: *β* = −1.16, *P* < 0.001) (Fig. 2A). In presymptomatic mutation carriers, a trend of statistical significance was found between having lower DTI-ALPS and closer expected years of onset (*β* = −9.94, *P* = 0.096) (Fig. 2B).

In all mutation carriers, lower DTI-ALPS was associated with higher plasma NfL, although with a trend of statistical significance (*β* = −0.28, *P* = 0.063) (Fig. 2C). No significant association was observed between DTI-ALPS and plasma GFAP (*β* = −0.10, *P* = 0.379) (Fig. 2D).

### Association between the glymphatic system status at baseline and longitudinal worsening in disease severity

**Figure 3:**
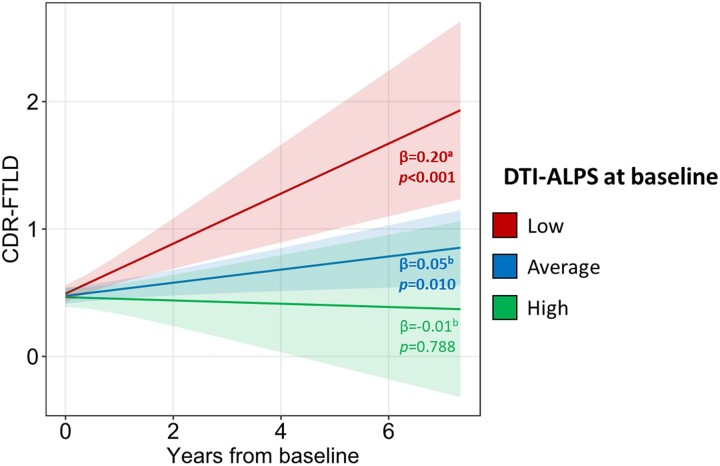
**Association between DTI-ALPS at baseline and longitudinal worsening in disease severity.** DTI-ALPS, diffusion tensor image analysis along the perivascular space; CDR-FTLD, CDR® Dementia Staging Instrument plus NACC behaviour and language domains. Baseline DTI-ALPS scores of mutation carriers were converted into *Z*-scores using mean and standard deviation of healthy controls, and mutation carriers were classified as having ‘high’ (*Z*-score > 1), ‘average’ (*Z*-score between −1 and 1) or ‘low’ (*Z*-score < −1) baseline DTI-ALPS. Statistics values: *χ*^2^(2) = 10.39, *P* = 0.006. *Post hoc* comparisons: ^a^ > ^b^.

All mutation carriers with low (*β* = 0.20, *P* < 0.001) or average (*β* = 0.05, *P* = 0.010) DTI-ALPS at baseline showed worsening in disease severity, while those with high DTI-ALPS remained stable over time (*β* = −0.01, *P* = 0.788) (Fig. 3). Worsening in disease severity was faster in mutation carriers with low DTI-ALPS at baseline than in those with average (*P* = 0.009) or high (*P* = 0.006) DTI-ALPS at baseline.

## Discussion

The glymphatic system represents a potential novel therapeutic target for the treatment of neurodegenerative disorders, while innovative non-invasive imaging techniques can quantify glymphatic system *in vivo* to elucidate pathophysiology. In the present study, we reported that glymphatic system, as indirectly measured by DTI-ALPS index, is impaired in genetic frontotemporal dementia. In particular, glymphatic system was significantly impaired in symptomatic *C9orf72* expansion carriers as compared with healthy controls, but the same trend was also observed in other mutation subgroups. Furthermore, glymphatic system dysfunction was not an early event and was undetectable in the presymptomatic disease stages, although it is unclear if this reflects late emergence of glymphatic deficits or limited sensitivity of non-invasive methods.

We have used the non-invasive MRI index, the DTI-ALPS, as a measure of diffusivity along the perivascular space. This we interpret as a measure of glymphatic function, at least for what concerns its perivascular component.^18^

Recent work has consistently reported impairment of DTI-ALPS index as a proxy of glymphatic system function in neurodegenerative disorders and in sporadic frontotemporal dementia as well.^10-14,17,20,21^ This confirms and extends previous research data, claiming that glymphatic system impairment is not disease-specific but reflects brain distress and neurodegeneration.^34^ In this perspective, a preserved glymphatic system might be protective against disease progression and clinical worsening, even in carriers of frontotemporal dementia-related mutations, while an altered glymphatic system might be associated with worse clinical outcomes over time.

These results support the hypothesis that brain clearance system continues to decline with disease progression, leading to increased tau or TDP-43 pathological burden, further raising the protein concentration to a level that favours aggregation. Glymphatic system dysfunction may participate in the symptom worsening of genetic frontotemporal dementia through its role in the clearance of pathogenetic proteins, autoimmune cell infiltration and promotion of inflammatory activation.^17^

In this view, we also reported a mild negative association between glymphatic function and expected age at onset of symptoms and between glymphatic function and markers of neurodegeneration, such as NfL,^25,26,33^ although not statistically significant.

Interestingly, longitudinal assessment of participants in the GENFI cohort allowed us to demonstrate that glymphatic function may clearly predict disease progression over time, suggesting that DTI-ALPS index may be regarded as marker of progression, possibly predicting transition towards progressive stages.

These results suggest the potential of glymphatic change as a therapeutic target in frontotemporal dementia, counteracting disease progression by enhancing the brain endogenous waste clearance pathways. The evidence for specific interventions remains indirect, e.g. that sleep deprivation can suppress brain clearance via the glymphatic dysfunction^35-37^ and consequently behavioural or pharmacological interventions that preserve night sleep could be considered. Although the study of sleep disturbances in FTD is in its infancy, there is emerging evidence that hypothalamic dysfunction, manifesting as disturbances in sleep, is an integral component of neurodegeneration in frontotemporal dementia.^38^ In addition to sleep, another potential molecular target could be represented by aquaporin-4, a key protein involved in the glymphatic system function,^39^ already demonstrated to be impaired in FTD patients in the GENFI cohort.^40^ In the near future, both pharmacological strategies or non-pharmacological approaches (e.g. diet) might be considered to modulate acquaporin-4 metabolism.^41-43^

We acknowledge that this study entails some limitations. First, the DTI-ALPS index was calculated based on the diffusion of perivascular space and may not be directly equivalent to the glymphatic function, although it has been shown to be reliable and has been widely applied in multiple diseases. In addition, in line with more recent studies on this topic, we did not use susceptibility-weighted imaging for ROI placement.^17,32^ Second, we assessed DTI-ALPS index of the whole brain rather than a regional function measurement, even though we tested left and right hemispheres with no substantial differences across clinical phenotypes. Third, we did not take into account possible additional confounders, such as white matter hyperintensities.

Notwithstanding these limitations, we believe that by using a non-invasive diffusion-based imaging approach, as a proxy of the glymphatic system function, we have successfully demonstrated that glymphatic system is impaired in the symptomatic stages of genetic FTD, irrespective of the clinical syndrome or neuropathological features. Moreover, glymphatic system impairment was associated with disease stage and predicted its clinical progression. The glymphatic system may therefore represent a novel potential target for future treatment approaches in genetic FTD.

## Supplementary Material

fcae185_Supplementary_Data

## Data Availability

Data will be shared according to the GENFI data sharing agreement, after review by the GENFI data access committee with final approval granted by the GENFI steering committee.

## Appendix I: Coinvestigators of GENFI Consortium

**Table:**

Author
David L. Thomas
Emily Todd
Caroline V. Greaves
Jennifer Nicholas
Kiran Samra
Rhian Convery
Carolyn Timberlake
Thomas Cope
Timothy Rittman
Andrea Arighi
Chiara Fenoglio
Elio Scarpini
Giorgio Fumagalli
Vittoria Borracci
Giacomina Rossi
Giorgio Giaccone
Giuseppe Di Fede
Paola Caroppo
Sara Prioni
Veronica Redaelli
David Tang-Wai
Ekaterina Rogaeva
Miguel Castelo-Branco
Morris Freedman
Ron Keren
Sandra Black
Sara Mitchell
Christen Shoesmith
Robart Bartha
Rosa Rademakers
Jackie Poos
Janne M. Papma
Lucia Giannini
Rick van Minkelen
Yolande Pijnenburg
Benedetta Nacmias
Camilla Ferrari
Cristina Polito
Gemma Lombardi
Valentina Bessi
Michele Veldsman
Christin Andersson
Hakan Thonberg
Linn Öijerstedt
Vesna Jelic
Paul Thompson
Tobias Langheinrich
Albert Lladó
Anna Antonell
Jaume Olives
Mircea Balasa
Nuria Bargalló
Sergi Borrego-Ecija
Ana Verdelho
Carolina Maruta
Tiago Costa-Coelho
Gabriel Miltenberger
Alazne Gabilondo
Ana Gorostidi
Jorge Villanua
Marta Cañada
Mikel Tainta
Miren Zulaica
Myriam Barandiaran
Patricia Alves
Benjamin Bender
Carlo Wilke
Lisa Graf
Annick Vogels
Mathieu Vandenbulcke
Philip Van Damme
Rose Bruffaerts
Koen Poesen
Pedro Rosa-Neto
Serge Gauthier
Agnès Camuzat
Alexis Brice
Anne Bertrand
Aurélie Funkiewiez
Daisy Rinaldi
Dario Saracino
Olivier Colliot
Sabrina Sayah
Catharina Prix
Elisabeth Wlasich
Olivia Wagemann
Sandra Loosli
Sonja Schönecker
Tobias Hoegen
Jolina Lombardi
Sarah Anderl-Straub
Adeline Rollin
Gregory Kuchcinski
Maxime Bertoux
Thibaud Lebouvier
Vincent Deramecourt
Beatriz Santiago
Diana Duro
Maria João Leitão
Maria Rosario Almeida
Miguel Tábuas-Pereira
Sónia Afonso
