# Supplementary material for: Impaired glymphatic system in genetic frontotemporal dementia: a GENFI study
Source: Brain Commun. 2024 Jun 14;6(4):fcae185. doi: 10.1093/braincomms/fcae185 (PMC11249959; doi:10.1093/braincomms/fcae185)
Supplement: fcae185_Supplementary_Data [file fcae185_supplementary_data.docx]

**SUPPLEMENTARY MATERIAL**

**Supplementary Table 1. List of GENFI sites and number of included subjects for each site.**

| **Site** | **Type of**  **scanner** | **Non carriers – Young** | **Non-carriers – Old** | **Pre**  **symptomatic** | **Symptomatic** | **Dicrections**  **number** |
| --- | --- | --- | --- | --- | --- | --- |
| GENFI_01 | Siemens Trio | 4 | 3 | 11 | 5 | 64 |
| GENFI_02 | Siemens Prisma | 0 | 0 | 0 | 4 | 64 |
| GENFI_03 | Philips Achieva | 5 | 8 | 33 | 4 | 61, 64 |
| GENFI_04 | Siemens Prisma,  GE Discovery | 5 | 2 | 12 | 1 | 64 |
| GENFI_05 | Siemens Trio,  Siemens Prisma | 3 | 1 | 5 | 10 | 64 |
| GENFI_06 | Siemens Skyra | 1 | 2 | 15 | 12 | 64 |
| GENFI_07 | Siemens Trio | 5 | 2 | 26 | 5 | 64 |
| GENFI_08 | Siemens Skyra | 6 | 1 | 13 | 0 | 64 |
| GENFI_09 | Siemens Trio | 8 | 2 | 13 | 2 | 64 |
| GENFI_10 | Siemens Prisma | 4 | 2 | 5 | 0 | 64 |
| GENFI_11 | Siemens Prisma | 1 | 0 | 2 | 3 | 64 |
| GENFI_12 | Siemens Skyra | 2 | 1 | 8 | 10 | 64 |
| GENFI_13 | Siemens Trio | 0 | 2 | 9 | 7 | 64 |
| GENFI_14 | Siemens Trio | 2 | 0 | 5 | 0 | 64 |
| GENFI_15 | Siemens Trio | 0 | 0 | 7 | 1 | 64 |
| GENFI_16 | Siemens Trio | 2 | 1 | 13 | 2 | 64 |
| GENFI_17 | Siemens Trio,  Siemens Prisma | 1 | 6 | 22 | 26 | 64 |
| GENFI_18 | GE Signa | 1 | 0 | 0 | 0 | 64 |

**Supplementary Figure 1. Region of interest (ROI) anatomical placement for DTI-ALPS.** Graphical representation of ROI placement on standard fractional anisotropy (FA) template (FSL_HCP1065_FA standard space). Two 5 mm spherical ROIs were placed *a priori* in the projection (red fibers) area and two 5 mm spherical ROIs in the association (green fibers) area at the level of the lateral ventricle bodies, on the left and right hemispheres.

**
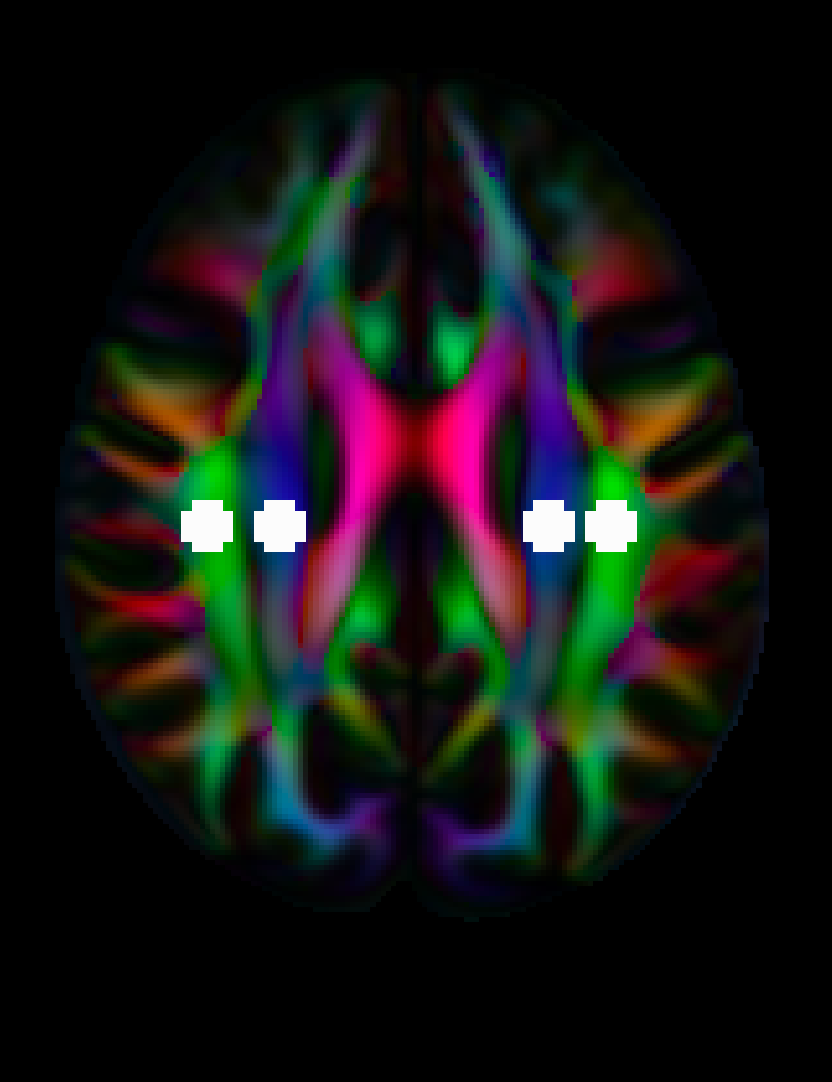
**
